# Mobile Tele-Mental Health: Increasing Applications and a Move to Hybrid Models of Care

**DOI:** 10.3390/healthcare2020220

**Published:** 2014-05-06

**Authors:** Steven Richard Chan, John Torous, Ladson Hinton, Peter Yellowlees

**Affiliations:** 1 Department of Psychiatry, University of California, Davis, 2230 Stockton Blvd., Sacramento, CA 95817, USA; E-Mails: ladson.hinton@ucdmc.ucdavis.edu (L.H.); peter.yellowlees@ucdmc.ucdavis.edu (P.Y.); 2 Harvard Longwood Psychiatry Residency Training Program, Boston, MA 02215, USA; E-Mail: jtorous@bidmc.harvard.edu

**Keywords:** telemedicine, telepsychiatry, telemental health, smartphone, mobile, technology, videoconferencing, ecological momentary assessment, mental disorders, psychiatry

## Abstract

Mobile telemental health is defined as the use of mobile phones and other wireless devices as applied to psychiatric and mental health practice. Applications of such include treatment monitoring and adherence, health promotion, ecological momentary assessment, and decision support systems. Advantages of mobile telemental health are underscored by its interactivity, just-in-time interventions, and low resource requirements and portability. Challenges in realizing this potential of mobile telemental health include the low penetration rates of health applications on mobile devices in part due to health literacy, the delay in current published research in evaluating newer technologies, and outdated research methodologies. Despite such challenges, one immediate opportunity for mobile telemental health is utilizing mobile devices as videoconferencing mediums for psychotherapy and psychosocial interventions enhanced by novel sensor based monitoring and behavior-prediction algorithms. This paper provides an overview of mobile telemental health and its current trends, as well as future opportunities as applied to patient care in both academic research and commercial ventures.

## 1. Introduction

Telemedicine—a term often interchanged with telehealth—encompasses “videoconferencing, the Internet, store-and-forward imaging, streaming media, and terrestrial and wireless communications” [1]. Telemedicine is especially useful for patients who cannot access specialists in their community, patients from differing cultural and linguistic backgrounds, patients of lower socioeconomic status, patients who prefer to receive care at home instead of in hospitals, institutionalized populations such as prisoners or the elderly, and patients who need special interpreting skills that are not available locally [2]. Patients also use telemedicine as an adjunct to their treatment, participate in online self-help or support groups, and keep in touch with their healthcare providers while traveling.

Providing mental healthcare through real-time videoconferencing comprises the bulk of research in telemental health technologies as this modality is most similar to traditional face-to-face mental healthcare practices [3]. Studies have shown that telemental health is effective and increases access to care. Videoconferencing, in particular, is as effective as in-person care “for most parameters such as feasibility, outcomes, age, and satisfaction with a single assessment and consultation or follow-up use” [4].

However, telemental healthcare also encompasses other modalities, such as “online therapy, eHealth, mobile technology, and health information technology” [3]. The use of mobile technologies, in particular, is rapidly evolving within the field of tele-mental health. Mobile health, or mHealth, is conducted on “mobile phones, patient monitoring devices, personal digital assistants (PDAs), and other wireless devices” [5].

This article provides an overview of mobile telemental health as it is being used for patient care. For the purposes of this paper, we will focus on smartphone devices. We will exclude personal digital assistant (PDA) devices that do not include a network connection, and cellular phones and early smartphones that do not include modern smartphone capabilities including advanced processing power, graphics, ability to run mobile applications, and interactive capabilities such as touchscreens.

## 2. Potential Advantages of Smartphones in Telehealth

Smartphones are well suited for health information dissemination and are increasingly being used as devices for health service delivery. As of 2013, 61 percent of United States mobile phone subscribers owned smartphones [6] with higher-end capabilities such as larger displays, GPS and accelerometer sensors, and higher processing power than low-end cell phones. These typically run on the iOS or Android platforms [7]. Worldwide, there are more than 3.2 billion unique mobile users, with an increasing number of wireless connectivity subscriptions [8].

According to the Pew Research Center 2012 report, over 31 percent of cell phone users have used their devices to look up health information with cell phone owners who are Latino or African American more likely to gather health information through their smartphones. And caregivers—who are critical to a psychiatric patient’s network—are also more likely to look up health information. This presents an opportunity for mental health applications targeting caregivers and minorities.

Mobile devices—in the context of healthcare—can be used for treatment monitoring and adherence, appointment reminders, community mobilization, health promotion, health surveys and surveillance, patient monitoring, decision support systems, and patient recordkeeping, as well as for the delivery of direct patient services via audio or video systems [5]. Software applications for providing or monitoring mental healthcare on smart phones are being created around the world [9,10]. An advantage of mobile phones is their ability to provide just-in-time interventions using “push” processes without requiring effort on the part of the patient [11]. Because of these devices’ portability and low power requirements, they are ideal for low- to middle-income countries that have less established communications and electrical infrastructure, and can allow impoverished individuals and communities relatively inexpensive access to the Internet. Mobile devices thus have an advantage with their lower cost *versus* that of traditional videoconferencing infrastructure. A recent paper summarizing all telemental health cost studies since 1998 mentions fixed costs for traditional real-time telepsychiatry infrastructure can go up to the tens of thousands of U.S. dollars [4].

Patients with certain conditions may also do better with mobile devices instead of desktop computers. Younger generations of patients are accustomed to not only using mobile devices but keeping them with them nearly all of the day [12]. Previous work has shown that telepsychiatry may be better used for particular patients—paranoid patients, young children, and people with severe social anxiety and autism—than using face-to-face in-person services [13]. Researchers and clinicians can potentially extend these advantages of telepsychiatry to take advantage of the ubiquitous nature of mobile devices and provide a more intimate, realistic and quantifiable assessment of patients’ lives.

## 3. Challenges to Adopting Mobile Devices in Telehealth

### 3.1. Health Applications Have Low Penetration Rates

Although smartphone use has pervaded mainstream culture, health applications are less pervasive. In the United States, Pew has reported that while one in five smartphone owners had a health application installed on their phone, less than 1 percent of health application users had an application to track mood or sleep, and only 2 percent used an application for medication tracking, alerts, and management [14]. The low healthcare penetration rates for application usage mirror historically slow rates of technology adoption within the healthcare industry and signify the need for considerable work on change management with both providers and patients to increase the rate of penetration.

Other factors that exacerbate penetration rates include unequal access to mobile devices. Different age groups, for instance, have lower rates of adoption of mobile devices [3]. Although smartphones, and cellular data plans are relatively cheap, low socioeconomic status still prevents some patients from being able to afford them. The use of technology itself is dependent on the patient’s technological aptitude and understanding how to effectively use digital information, and thus, e-health literacy is paramount, and this still affects a proportion of the population who are older than those “digital natives” who have lived their whole lives with the Internet [15].

### 3.2. Current Published Research Tends to Evaluate Older Technologies

From a research perspective, most clinical trials to date involving mobile telemedicine focused primarily on text messaging as a method of remote communication, assessment, and intervention. Text messaging for appointment reminding, for instance, has been shown to have modest benefits with no difference being found between text messaging reminders compared with other reminders via post or phone call [16] There are fewer published clinical trials devoted to evaluating smartphone applications, despite more than a decade of the smartphone’s existence. A lack of robust scientific research base may preclude adoption of these applications as insurance organizations require thorough assessment of technologies before determining they are eligible for coverage. The Centers for Medicare and Medicaid Services (CMS), for instance, adopts a Medicare National Coverage Process that includes technology assessments conducted by staff reviewing evidence through systematic reviews of medical and scientific articles and study criteria “to assess its validity ... clinical relevance ... and weight (magnitude of effect)” [17].

The recent incorporation of front-facing cameras in smartphones and tablet computers has enabled video calls using freely available consumer software for mobile phones, such as Microsoft’s Skype, Google Hangouts and Apple’s FaceTime. Despite the widespread availability of such software, and the fact that the iPhone has had such hardware since June 2010 with the iPhone 4, to date, only two published studies have tested synchronous smartphone videoconferencing for telemedicine, with somewhat mixed results.

Mayo Clinic researchers described a telestroke service using built-in FaceTime videoconferencing software on Apple iPhone 4 systems on a secure internal WiFi network compliant with the United States Health Insurance Portability and Accountability Act (HIPAA) [18]. This study found that remote neurologists could perform National Institutes of Health Stroke Scale (NIHSS) assessments on the iPhone 4 with good correlations to bedside vascular neurologists’ scores. Researchers at the National Center for Telehealth and Technology successfully demonstrated FaceTime as a usable tool in conducting interviews from the United States to military installations in Asia; however, network connection problems caused video quality to degrade [19].

A number of studies have been undertaken on mobile applications, especially when used for monitoring, and these will be reviewed later.

### 3.3. Current Mobile Health Studies Need Improved, Newer Research Methodologies

Mobile health clinical trials, in general, have been said to suffer from lack of standardization, lack of sound methodology, and lack of data interoperability and systems integration [20]. Most pilot projects have been described as employing a “scatter-shot approach” that creates the “equivalent of black boxes,” requiring patients to use multiple applications or methods to manage their health.

Researchers at the mHealth Summit 2013 conference in Maryland emphasized the need for different research methods and approaches [21,22,23]. Research trials should employ more efficient, adaptive intervention study techniques such as the Sequential Multiple Assignment Randomized Trial (SMART), a type of randomized trial best used for technology interventions that use multiple components [24]. This would allow, for instance, researchers to study the efficacy of individual components and the sequence of such components, such as offering SMS text messages with supportive messaging, SMS text messages with directive messaging, smartphone notification alerts, or telephone calls, or a combination thereof. One researcher cited the need to look at habit and timing in mobile health interventions, *versus* traditional evaluation of cognition via surveys, questionnaires and other traditional psychological evaluation methods [25]. Others noted that researchers need to work with other disciplines to transcend the barriers of data interoperability and standardization—such as how particular interventions are defined, how data is processed, and how to normalize data from wildly different wearable sensors.

Despite these challenges, there is optimism for smartphones’ role in healthcare. Other factors driving this optimism include the current unsustainable healthcare expenditures in the U.S. along with a need for personalized, more precise assessments and interventions [8]. A number of commercial ventures such as American Well have commenced physician visits via iOS and Android devices to consumers for video consults [26] despite criticism over potential problems with coordinated care and inadequate diagnosis of diseases like strep throat [27]. Many challenges continue to face mobile health: technical issues, cost, clinical appropriateness and validity, and ethical issues. These challenges are also relevant to tele-mental health.

## 4. Taking Advantage of Mobile Devices for Mental Health

The advantages of mobile telemental health are similar to those applied to mobile telemedicine in general: smartphones’ portability allows them to be used independently of a particular location; they are inexpensive *versus* traditional desktop and laptop computer-based solutions; and they can be used for immediate context-aware interventions [28].

Interestingly the majority of patients with mental illness own mobile devices. Of 1592 individuals with serious mental illness in metropolitan Chicago surveyed in 2013, 72 percent owned a mobile device, including not just smartphones, but also “mobile phones ... and devices that enable text-messaging for people with hearing impairment.” Common uses included phone calls, text messaging, and Internet use [29]. A recent survey we conducted at a university-affiliated outpatient psychiatry clinic demonstrated that 70 percent of 100 patients surveyed owned a smartphone, and over 50 percent of those owning a smartphone were willing to download a mobile application to monitor their mental health [30]. In fact, respondents expressed more interest in using a mobile application than text messaging.

There are an enormous number of applications with more than 3000 mobile device applications, including mood trackers and CBT applications in both Apple’s App Store for iOS devices and Google Play for Android device [31]. Table 1 presents the number of available applications, derived from a basic search on Google Play using the AppBrain website and the iTunes App store website as of 14 January 2014. The Android and Apple operating systems were selected as since 2013, over 90 percent of smartphones sold in the United States are Android- or iOS-based [32].

**Table 1:** Statistics of applications on the Google Play platform via AppBrain, and Apple’s App Store.

Search terms	Google Play January 2014	iOS App Store January 2014
depression	1615	586
anxiety	1269	775
schizophrenia	67	20
bipolar	151	90
psychiatry	168	149
alcoholism	1911	146

Mobile tele-mental health applications have thus far fallen under three categories as below, most of which involve them being used as an adjunct to in-person care, or as part of a hybrid care environment where the patient receives care both in person and online:

■ a communications medium for psychotherapy
■ an extension of psychotherapy
■ a psychosocial intervention using novel monitoring approaches

### 4.1. A Videoconferencing Medium for Psychotherapy

Mental health, an area of healthcare that relies much more heavily on face-to-face interactions than other medical specialties, can take advantage of modern-day mobile device audio-video capabilities where the smaller resolution of mobile devices is an advantage. A study comparing facial affect recognition in teleconferencing found counterintuitively that a horizontal resolution of 280 lines commonly used in VHS-grade videotapes offered improved facial affect recognition over 480 lines in Betacam-grade videotapes. Thus, a lower resolution, especially on a smaller screen, may well be better for facial recognition and the understanding of facial affect than when seen on a larger screen [33].

Although few clinical studies have been published, there are a number of clinical services currently in operation. Veterans and military service members can already use live video sessions with licensed mental health professional volunteers for counseling through the Give an Hour nonprofit organization [34]. The service uses the HIPAA-compliant Google Helpouts videoconferencing platform for questions and answers and works on Android devices [35].

Dedicated videoconferencing services on mobile devices also exist in the commercial sector. One service, American Well, features Android and iOS applications [26] on the ValueOptions national behavioral healthcare network [36]. HealthLinkNow offers video sessions over webcam-equipped mobile devices [37]. The 1docway service connects psychiatrists with patients through a web application compatible with the Chrome browsers on Android. Talksession uses HIPAA-compliant mobile technology for remote psychotherapy sessions, particularly to address younger patients who rely more on mobile devices [38]. Another company, Breakthrough, plans to provide video psychotherapy from mobile devices in 2014 [39].

The Google Helpouts video service provides help for general leisure topics such as cooking, art, and gardening, as well as concierge medical services. We found 22 individual psychologists offering to provide psychotherapy through the mobile-enabled video service as of December 2013 [40].

### 4.2. As an Extension of Psychotherapy

Mobile devices can be used to extend the practice of psychotherapy, from cognitive behavioral therapy to addiction medicine, and many descriptive symptom gathering and monitoring applications are available which may also be used as electronic diaries for patients to self-monitor their activities and moods, and encourage therapy homework [28]. Gathering a patient’s “symptoms, affect, behavior, and cognitions close in time to experience” is known as *ecological momentary assessment* (EMA) [41]. A feasibility study demonstrated that a prototype app, iHabit for iOS, can be used as an EMA tool on smartphones to gather patient responses to question prompts during researcher-specified hours of the day [42]. Mobile devices can equally extend wrap-around case management services, allowing clinicians to intervene at the immediate moment before a patient relapses on their alcohol, smoking, or gambling addiction.

The use of mobile devices for these purposes in mental health has been driven by military and veteran needs. The National Center for Telehealth and Technology, and the U.S. Department of Veterans Affairs, have, since 2011, piloted and released applications geared towards caregivers, healthcare providers, and patients [43]. Their applications, in particular, aid in post-traumatic stress disorder (PTSD) symptom reporting with direct integration with their electronic medical record (EMR) [44]. Their CBT-i Coach application acts as an electronic diary of patients’ sleep combined with cognitive behavioral techniques and education [45]. Others include PE Coach to support prolonged exposure (PE) treatment for PTSD [46] and the Stay Quit Coach to aid in tobacco cessation [47].

In the general population, several tobacco cessation applications have also taken advantage of smartphone capabilities, such as the National Cancer Institute’s Quitpal application that includes reminders, video diaries, and social networking site connections [48]. Researchers in Sweden have also incorporated behavioral activation—a psychological treatment based on learning theory—and mindfulness techniques in a pair of smartphone applications for patients with depression*.* [9]. Researchers in Australia have also studied the effects of a suicide prevention application in indigenous Australian youths [10].

### 4.3. As a Psychosocial Intervention with Novel Monitoring Approaches

Early work indicates that smart applications are feasible as an intervention across numerous mental health conditions. Within tobacco cessation, smartphones can play a significant role as they can be used “when and where they are needed” as part of a hybrid behavioral intervention combining in person and online strategies [48]; we believe this claim applies to many areas of mental health especially those involving addictions.

For depression, one paper reported feasibility, preliminary evidence of efficacy, and a high level of satisfaction among among seven patients who used a smartphone application for eight weeks [49]. Addressing schizophrenia, one group demonstrated the feasibility of ambulatory monitoring six times per day in 36 patients with schizophrenia using a smartphone application [50]. Another group has proposed a smartphone application for smoking cessation [51] while another demonstrated the acceptability and feasibility of utilizing a smartphone’s camera and internet capabilities as an intervention for smoking cessation but did not utilize an actual application [52]. For alcohol use disorders, one group reported high rates of sustained use of a mobile application designed for preventing relapses among 349 patients during the first four months of an ongoing study [53].

The recent incorporation of sensors in smartphones has opened up the possibility of using machine learning prediction for intelligent assessments and intervention. Ecological momentary interventions by Burns *et al.* [49] incorporated in the Mobilyze smartphone application helped as an aid for major depressive disorder. The application based its predictions of a patient’s mood, cognitive state, emotions, and environmental and social context on more than 38 phone sensor values, such as global positioning system status, ambient light, and recent calls [49]. Similar technologies have been incorporated into the Purple Robot open-source Android sensor framework for other researchers and developers to use [54]. Integration of sensors to predict mood is theorized to predict schizophrenia relapse via individualized early warning signs such as isolation, lack of movement, or unusual behavior in a future clinical trial by Dartmouth University researchers [29]. Other areas to be explored are the potential for mobile devices to strengthen self-management and behavioral action, especially in patients with other non-psychiatric disorders. The SPARX video game, for instance, currently employs cognitive behavioral therapy to target depression in adolescents [55], and the smartphone application and online game will expand to cover co-morbid conditions such as cancer, diabetes, and asthma [56]. In a randomized controlled trial, found to clinically reduce depression, anxiety, and hopelessness and “was at least as good as” face-to-face counseling by clinical psychologists in New Zealand [55].

## 5. Challenges for Mobile Tele-Mental Health

All the potential barriers to mobile telemedicine also apply to mobile mental health: network and device security, network and service availability, reliability, and efficacy of assessments and interventions, and patient adherence to mobile applications. A recent review on mental health intervention technologies even notes that the definition of *adherence* can be elusive—for instance, number of logins, number of activities completed, amount of time spent—and whether measuring such statistics would appropriately indicate the success or failure of the intervention [57]. A patient who stops using an application may indicate that the patient has already received enough benefit from the application, or the opposite: that they were not sufficiently engaged and the application has failed in its intent. Mohr *et al.* [57] further suggests that improving usability, adding human support, and adding game elements could boost patient engagement.

Importantly, given that the use of mobile devices in mental health is still in its infancy, best practices for behavioral interventions have not yet been standardized or thoroughly researched. For instance, a recent review in 2013 noted that, out of 5464 abstracts examining the effects of mental health mobile applications, only eight papers described five applications targeting depression, anxiety, and substance use. Two of these five applications were available for public download on application stores. Moreover, the majority of applications lacked scientific evidence about their efficacy, and no studies examined the long-term efficacy of mental health applications. The quality of studies analyzed was low, which the review authors note may be biased towards positive results. They note that research is particularly weak in sleep disorders, anxiety disorders, and smoking cessation [31].

Of the mental health applications available for Android, iOS, and Windows Phone platforms, Donker *et al.* [31] concluded that less than 1 percent of the commercially available applications included evidence-based practices, citing a conclusion by another review that the development of mHealth applications was driven by commercial intent rather than by research and science [58]. Most of these applications may not survive commercially and may drop out of existence, being replaced by others, and evaluating the efficacy and appropriate use of such applications is an insurmountable task for the harried healthcare provider but is something that over time is necessary for those applications that receive significant patient and provider uptake.

## 6. Conclusions

Mobile devices can be an integral part of assessment and intervention for psychiatric disease as we increasingly move to a hybrid model of mental healthcare delivery with larger numbers of patients receiving both in person and in online interventions for diagnosis, therapy, and monitoring. Most of the innovative cutting-edge activity is occurring in the commercial sector; however, few of these efforts—particularly applications—are being clinically validated. Research studies on best practices and clinical validity are increasing and as of November 2013, ClinicalTrials.gov’s database of behavior and mental disorder clinical research studies contained 102 open studies involving smartphones [59]. Organizations such as the American Telemedicine Association, the Healthcare Information and Management Systems Society, and the American Medical Informatics Association are leading efforts in providing mHealth standards and guidelines while a number of professional societies are addressing practice standards.

Mobile health is fast evolving, encompassing new devices, sensors, and interfaces, and mental health is at the forefront of this movement. One commentary has predicted a convergence of the telemedicine and mobile health fields, estimating that up to 50 percent of all healthcare transactions in the healthcare industry by 2020 will be “electronically out-sourced,” and that 25 percent of all patient encounters with healthcare professionals could be by mHealth, utilizing smartphones or smart wrist watches [60]. Mobile processes and smart devices will inevitably continue their foray into traditional healthcare practices so that “hybrid” practices of care become the norm for most patients over time. For this to occur successfully and for this approach to hybrid mobile healthcare to be of high quality we will require new research methods for evaluating mobile health efficacy and best practices and for integrating mobile health into optimal, clinical workflows.
